# Biomarkers in precision therapy in colorectal cancer

**DOI:** 10.1093/gastro/got022

**Published:** 2013-08-23

**Authors:** Marlies S. Reimers, Eliane C.M. Zeestraten, Peter J.K. Kuppen, Gerrit Jan Liefers, Cornelis J.H. van de Velde

**Affiliations:** Department of Surgery, Leiden University Medical Center, Leiden, The Netherlands

**Keywords:** colorectal cancer, biomarkers, treatment, prognosis

## Abstract

Colorectal cancer (CRC) is the most commonly diagnosed cancer in Europe. Because CRC is also a major cause of cancer-related deaths worldwide, a lot of research has been focused on the discovery and development of biomarkers to improve the diagnostic process and to predict treatment outcomes. Up till now only a few biomarkers are recommended by expert panels. Current TNM criteria, however, cause substantial under- and overtreatment of CRC patients. Consequently, there is a growing need for new and efficient biomarkers to ensure optimal treatment allocation. An ideal biomarker should be easily translated into clinical practice, to identify patients who can be spared from treatment or benefit from therapy, ultimately resulting in precision medicine in the future. In this review we aim to provide an overview of a number of frequently studied biomarkers in CRC and, at the same time, we will emphasize the challenges and controversies that withhold the clinical introduction of these biomarkers. We will discuss both prognostic and predictive markers of chemotherapy, aspirin therapy as well as overall therapy toxicity. Currently, only mutant KRAS, mutant BRAF, MSI and the Oncotype DX® Colon Cancer Assay are used in clinical practice. Other biomarker studies showed insufficient evidence to be introduced into clinical practice. Divergent patient selection criteria, absence of validation studies and a large number of single biomarker studies are possibly responsible. We therefore recommend that future studies focus on combining key markers, rather than analysing single markers, standardizing study protocols, and validate the results in independent study cohorts, followed by prospective clinical trials.

## INTRODUCTION

Colorectal cancer (CRC) is the most frequently diagnosed type of cancer in Europe and is one of the major contributors to cancer-related deaths worldwide [1, 2]. In 2008, 436 000 new cases of CRC were diagnosed in Europe and CRC was therefore the most frequently diagnosed cancer with 13.6% of all diagnosed cancers [1]. Worldwide, the percentage of total cancer burden contributable to CRC was 9.7%, with 1.23 million cases, following lung (1.61 million) and breast cancer (1.38 million) [3]. In Europe, CRC was responsible for 212 000 (12.2%) deaths in 2008, representing the second most common cause of death by cancer after lung cancer (19.9%) [4, 5]. Approximately 20–25% of patients with CRC already have metastatic disease at the time of diagnosis and 20–25% of patients will develop metastases during disease progression as well, resulting in a high 40–45% mortality rate [4, 5].

Studies aiming at optimizing the diagnostic process and treatment of this disease are increasing, which has probably caused CRC to be one of the most-studied and best-characterized processes of tumorigenesis. Through more biological knowledge of tumorigenesis in CRC, more emphasis on early detection and development of new and improved treatment regimens, mortality decreased by almost 5% over the last decade [2, 6, 7]. Unfortunately, overall mortality and morbidity rates in CRC still remain high [2].

Survival of CRC patients largely depends on the disease stage at diagnosis and varies widely between stages. Five-year survival for stage I is 93.6%, which drops drastically to 8.1% for stage IV patients [8]. Treatment of CRC comprises (radical) tumor resection and, depending on tumor stage, radio- or chemotherapy [9]. Treatment choices nowadays are influenced by the tumor, node and metastasis (TNM) classification of the Union for International Cancer Control (UICC) [10]. The TNM classification aims to provide an exact prediction system for prognosis, to guide therapy choices and to form an understandable and uniform ‘cancer language’ [11, 12]. Over the past decades, this TNM staging has changed continuously. In 2009, the seventh edition of the TNM stage was published, replacing the sixth edition from 2002 [13]. Regrettably, the seventh TNM edition did not provide greater accuracy in predicting colorectal cancer patients’ prognosis, but resulted in a more complex classification for daily clinical use [14].

Unfortunately, besides making tumor classification more complex over the past years, the TNM staging system has not provided the clinician with the optimal staging tool it was designed for. Furthermore, possible under-treatment or over-treatment of some patients groups might arise when using the TNM staging system for treatment allocation [10, 15–17]. Studies have shown that approximately 20–25% of patients with lymph node-negative stage II colon cancer will suffer from recurrent disease within 5 years of follow-up [18, 19]. These patients, also identified as high-risk stage II patients, might have benefitted from adjuvant therapy—which they did not receive as this was not recommended, based on their defined TNM stage. Therefore, the use of TNM stage falls short in daily clinical practice, especially in identifying high-risk stage II patients, and needs to be supplemented with additional biomarkers that can substantially improve the current staging and treatment allocation criteria. The American Society of Clinical Oncology’s Tumor Markers Expert Panel (ASCO TEMP-2006), the European Group on Tumor Markers (EGTM-2007) and the European Society of Medical Oncology (ESMO) have all reviewed the clinical applicability of widely studied biomarkers [20–23]. Interestingly, in spite of a tremendous amount of available literature on biomarkers in CRC, only a few biomarkers are nowadays used in daily clinical practice, such as KRAS, BRAF, MSI and the Oncotype DX® Colon Cancer Assay (Table 1). A possible explanation could be that most prognostic or predictive biomarkers are not validated in other (large) cohorts, or because there is lack of consensus in performing these studies, such as different antibodies used or different scoring methods, which makes their results incomparable [20]. Furthermore, the handling of tissues has been well recognized in contributing to assay variability and issues in assay validation [24]. Previously, a five-step program for the introduction of biomarkers in clinical practice was developed, with the first step being biomarker development in a preclinical, exploratory setting, followed by verification of this biomarker in a large retrospective study, validation and, finally, confirmation in a prospective, randomized, controlled trial [25].

In this review we aim to give an overview of the most-studied biomarkers in CRC and we will emphasize the challenges and controversies in studying these biomarkers. The main goal is to identify key biomarkers, which might have the potential to identify patients who can be spared from further treatment or for whom additional treatment is advised (prognostic biomarkers) and to identify those who will benefit from therapy (predictive biomarkers), ultimately resulting in the use of precision medicine in the future.

## PROGNOSTIC BIOMARKERS IN CRC

### Microsatellite instability

Most cancers of the colon and rectum display a phenomenon termed ‘genomic instability'. There are two forms of genomic instability that reflect different genetic pathways of tumorigenesis. One form, called microsatellite instability (MSI), refers to a clonal change in the number of repeated DNA nucleotide units in microsatellites caused by deletions or insertions, and appears in tumors with deficient mismatch repair (MMR) [26]. The molecular phenotype of MSI was first described in CRC by an independent research group, showing MSI as the hallmark of Lynch Syndrome, although it was not solely restricted to hereditary CRC [27]. The biochemical basis of this phenotype can be explained by strand-specific mismatch repair defects and was initially linked to germline mutations of the mismatch repair (MMR) gene hMSH2, followed by the identification of mutations in another MMR gene, hMLH1. Only a short period thereafter, mutations in PMS2 and hMSH6 were found in Lynch Syndrome, completing the biological background of this MSI phenotype [27, 28].

**Table I.** Biomarkers used in clinical practice

Biomarkers	Clinical use
KRAS	Identification of resistance to anti-EGFR moAB in metastatic CRC patients
BRAF	Identification of resistance to anti-EGFR moAB in metastatic CRC patients.
	Exclusion of Lynch Syndrome
MSI	Identification of Lynch Syndrome
Onco*type* DX colon cancer assay	Inform treatment planning in stage II and II colorectal cancer patients

Abbreviations: MSI = microsatellite instability.

Currently, there are a few clinical criteria for MSI testing in CRC to select potential Lynch Syndrome patients to be candidates for molecular MSI testing (Bethesda Guidelines): (i) three or more relatives with CRC across ≥2 generations with one first-degree relative and one with a cancer age below 50 years; (ii) CRC in a patient younger than 50 years of age; (iii) synchronous or metachronous CRC regardless of age; (iv) CRC with high-density tumor-infiltrating lymphocytes, Crohn’s-like lymphocytic reaction, mucinous/signet-ring differentiation or medullary growth pattern, patient age ≤60 years; (v) CRC in one or more first-degree relatives with CRC, with one cancer diagnosed in a patient with age ≤50 years and (vi) CRC in two or more first/second-degree relatives with CRC at any age [29, 30]. If there is a clinical suspicion of Lynch Syndrome, MSI testing with molecular screening and/or immunohistochemistry has been recommended by the ESMO Consensus Guidelines for management of patients with colon and rectal cancer and has been performed in clinical practice as well [23].

Contrary to Lynch Syndrome, a different mechanism causes the sporadic type of MSI to develop in CRC. This phenotype is associated with hMLH1 promoter hypermethylation, resulting in lack of hMLH1 expression and subsequently loss of mismatch repair system function [27]. If loss of hMLH1 is observed by MSI testing, somatic hypermethylation of the hMLH1 promoter should be considered. This sporadic type of MSI could be investigated through testing for a BRAF V600E mutation that is strongly associated with a sporadic origin or by analysis of hMLH1 promoter hypermethylation [31].

It has been shown that MSI CRC is associated with a better prognosis than non-MSI CRC [32–36]. Therefore MSI might also be introduced as a standard pathological assessment for patients not included in these guidelines. Unfortunately, results from studies have been equivocal concerning the proposed survival benefit and this has resulted in clinical testing for sporadic MSI CRC not being routine up to now [37–41]. There are several reasons that prevent the introduction of MSI testing into standard pathological assessment. First, the prognostic effect of MSI is better appreciated for disease-specific survival than for overall survival [42]. This could be explained by the better prognosis of young patients (<50 years) with MSI CRC, as probably more young patients are likely to have Lynch Syndrome [33]. To include older patients with sporadic CRC—who can die of other diseases—might result in loss of the positive prognostic effect of MSI in overall survival. Inclusion or exclusion of various age groups will likely influence the prognostic significance of MSI analysis. Nonetheless, several studies have reported a favorable outcome for patients with MSI [37–41]. Second, the survival advantage of MSI might also be the result of less distant metastases at diagnosis, lower prevalence of advanced stage tumors, high prevalence of early stages at diagnosis and for the largest part by younger Lynch Syndrome cases [43]. In conclusion, MSI is a marker for better clinical outcome, but appears to be more pronounced for Lynch Syndrome [42].

The reluctance to introduce routine testing of MSI in clinical practice is also based on several other factors. First, clinicians may not be aware of the criteria and conditions requiring genetic screening and mutational analysis. Second, availability of a standardized laboratory test might be inadequate. MSI testing in molecular pathology laboratories is becoming increasingly available, but requires expertise and experience in testing and interpretation. Nowadays, immunohistochemistry (IHC) shows high sensitivity and specificity in detecting MSI and could therefore offer a relatively cheap, easy to perform and universally available test for MSI, instead of a more complex polymerase chain reaction (PCR)-based MSI test [44]. Lastly, there are also socio-economic issues to resolve, such as ethical, legal and health care-related issues, before introducing MSI testing in clinical practice. Clearly, MSI has been used successfully in clinical practice for Lynch Syndrome diagnosis and also shows great clinical potential for routine testing of non-Lynch Syndrome CRCs, but more research must first be performed on MSI in sporadic as well as hereditary CRC to truly understand the better clinical outcome of MSI.

### KRAS

The RAS-family of oncogenes consists of three principal members, KRAS, HRAS and NRAS, which are all involved in tumor development [45]. KRAS is a proto-oncogene encoding a small 21 kD guanosine triphosphate/guanosine diphosphate binding protein modulating cellular proliferation and differentiation [46]. Active KRAS mutations are found in 35–42% of CRCs and are thought to occur early in CRC carcinogenesis. Almost 97% of all observed genetic events within KRAS are caused by seven different DNA base-pair substitutions within codon 12 and 13 of exon 2, resulting in an amino acid substitution in the protein [47]. KRAS mutation was associated with a significantly higher risk of recurrence in the QUASAR study compared with wild-type KRAS, but not in the PETACC-3 study [23, 47]. Other studies performed were also conflicting, with some finding a prognostic value of mutated KRAS alone, others finding this value concomitantly with mutated TP53 or PIK3CA and some reporting no prognostic value of mutated KRAS at all [47–51].

Differences in KRAS mutations at codon 12 and 13 may result in different biological and functional consequences that could influence the prognosis of CRC [52]. Initially, KRAS was found to be a strong prognostic factor in CRC, but this finding was later restricted to a codon 12 mutation, leading to a glycine to valine substitution (G12V) [53, 54]. Therefore, larger studies are required to confirm whether a specific mutation is responsible for a clinically relevant prognostic effect.

An important reason for the discrepancies between the studies could be the design of the individual studies. Data based on prospective analysis of a homogenous cohort, treated and followed according to the highest clinical standards, as performed in a registration trial, are more robust and reliable than those arising from similar sized meta-analyses or retrospective studies. Therefore, well-performed clinical trials should be used to validate results on KRAS in order to resolve discrepancies.

In conclusion, there are conflicts among current data, which do not support standard testing for KRAS mutations in clinical practice to identify patients with a worse prognosis and requiring more aggressive treatment. However, in a predictive setting, mutated KRAS has shown differentiation resistance to anti-EGFR monoclonal antibodies and since then has been used in clinic for this purpose.

### BRAF

The BRAF gene encodes a serine/threonine protein kinase belonging to the RAS-RAF-MEK-ERK kinase pathway regulated by KRAS protein activity and involved in CRC development [55, 56]. Nearly all oncogenic transformations of BRAF are the V600E mutations [57]. A lot of studies investigated and confirmed the potential adverse prognostic impact of BRAF mutations [47, 58–60]. Yokota *et al.* identified BRAF as an independent prognostic factor for survival in a retrospective cohort of 229 patients with advanced and recurrent CRC. Presence of this mutation was associated with a significantly higher risk of cancer-related death, independent of other confounding factors [60]. These findings were consistent with those of other recent studies using patients with both stage II and -III disease and with studies including all stages [47, 58, 59, 61].

The PETACC-3 and QUASAR studies showed no increased risk of relapse in stage II and -III CRC patients, but PETACC-3 did show a worse overall survival (OS), particularly in patients with MSI-L or MSS tumors [34, 47]. Two large retrospective studies are in accord with these findings [57, 59]. Samowitz *et al.* reported that the BRAF V600E mutation in MSS colon cancer was associated with a significantly poorer survival in stage II to IV colon cancer, but did not have an effect on the excellent prognosis of MSI tumors [57]. Some patients in these trials were treated with cetuximab after relapse. Patients with mutated BRAF may not have benefited from the survival advantage offered by this agent [62, 63]. Therefore, the prognostic relevance of mutated BRAF on OS may have been overestimated. However, the outcome of patients with CRC having BRAF mutations is worse than that of patients with wild-type BRAF CRC, independent of treatment with cetuximab [64], which further strengthens BRAF as a marker for a worse chance of survival.

### TP53

TP53 is a tumor suppressor gene on the short arm of chromosome 17, encoding a protein important in regulating cell division. P53 is normally expressed in case of DNA damage, resulting in growth arrest and apoptosis (programmed cell death) in rapidly dividing cells. In this way TP53 functions as a tumor suppressor gene by aborting growth of potentially malignant cells [65]. Mutations of the TP53 gene are detected in up to 85% of CRCs, usually occurring during the adenoma to adenocarcinoma transition [66]. Over the years, TP53 has been intensively studied as the genome guardian marker [67, 68]. The immunohistochemical expression may have prognostic value in patients with CRC. Higher expression has been shown in tumors with lymph nodal involvement and 5-year survival is lower for patients with positive p53 staining [69]. In normal cellular conditions, synthesis and degradation of p53 are tightly regulated and the expression level remains very low. In such conditions, p53 expression is generally not detectable by immunohistochemistry. Mutations in TP53 lead to disruption of normal TP53 function and accumulation of mutant p53 levels that are high enough to be detected by immunohistochemistry [70]. Lack of p53 staining with immunohistochemistry has been associated with wild-type TP53, indicating a functionally active TP53. High expression of p53 staining was associated with mutated TP53 [71]. However, there is still debate on the use of mutational analysis or immunohistochemistry as a reliable marker for p53 dysfunction. Lack of consensus on antibodies and scoring might possibly be responsible for this [72, 73]. Studies have shown that immunohistochemistry does not always match with mutation studies and that expression of mutant forms of p53 are not simply correlated to loss of TP53 function [70]. Cripps *et al.* reported that approximately 33% of the CRCs that do not show positive immunohistochemical staining of p53 do not have a detectable TP53 mutation [74]. Also, a scattered positive immunohistochemical staining of p53 might represent a functionally active, non-mutated TP53 gene and must therefore be analysed separately [73]. Most studies in the past, however, only analysed positive staining versus negative staining [69, 75–77]. Recently, Nyiraneza *et al.* investigated the value of immunohistochemistry of p53 in CRC [71]. In this study immunohistochemistry revealed three distinct staining patterns of p53 expression; complete negative staining associated with truncating TP53 mutations, diffuse overexpression associated with TP53 missense mutations and restricted overexpression associated with wild-type TP53. Furthermore, mutation analysis by Lopez *et al.* showed that TP53 mutations were only present in 79.6% of positively stained p53 tumors [70]. In 30.8% of the tumors with negative p53 staining, TP53 mutations were found as well, indicating no complete correlation between immunohistochemistry and mutation analysis based on RNA expression.

In summary, TP53 cannot so far be used as a prognostic marker. Lack of consensus on antibodies and scoring methods in immunohistochemical staining, lack of correlation between immunohistochemical overexpression and clinical data and discrepancies between immunohistochemistry and mutation analysis are responsible for conflicting results and are therefore important reasons for not justifying the use of TP53 in clinical practice.

### Apoptosis-related biomarkers

One of the most important hallmarks of cancers is their ability to evade programmed cell death or apoptosis [78]. During tumor development, tumor cells can be triggered by lymphocytes of the patient’s immune system, by accumulation of DNA damage, or by stress factors, like growth factor deprivation, to undergo apoptosis [79, 80]. The actual apoptotic cell death machinery—the part of the pathway responsible for the execution of apoptosis that results in the morphologic features characteristic for apoptosis—consists of a very complex cascade of interacting proteins. The key components are the caspases. Caspase-3 is activated at a point where the intrinsic and extrinsic apoptosis induction pathways converge. The level of activated caspase-3 should therefore give a reliable measure of ongoing apoptosis and is widely used in studies for detection of apoptotic cells [81]. M30, another commonly used marker specific for apoptotic epithelial cells, recognizes caspase-specific cleaved product of cytokeratin-18 [82].

Several publications have described the relevance of apoptosis to clinical outcome in CRC patients, with contradictory results [82–85]. Differences between these studies might have been caused by a different patient selection, a different method used and a different study design of these publications. There are also reasons to believe that differences exist as a result of microsatellite status of the tumor, location of the tumor in the bowel or biological differences between rectal and colon cancer [82–84, 86]. Dolcetti *et al.* reported a high frequency of apoptosis in MSI tumors [86]. Jonges *et al.* described a higher expression of cleaved caspase-3 expression in right-sided tumors [84]. In some rectal cancer studies, low expression of apoptosis was related to more local recurrences [82, 83]. In CRC, however, results were different, with high expression of apoptosis related to more local recurrence [84]. Reasons for these discrepancies are unclear. As most rectal cancers are MSS, microsatellite status might possibly explain these differences. Location of the tumor might also have an important influence on apoptosis. Recently, the Cancer Genome Atlas Network attempted to find biological differences between colonic and rectal cancer, but they only found differences in the anatomical tumor site with more hypermethylation in right-sided tumors, which might be explained by the different embryonic origins of the right-and left-sided tumors [87]. Additional research on apoptosis is warranted, with consideration of microsatellite status and the location of the tumor.

Furthermore, it might not be sufficient to study apoptosis on its own. A key factor in tissue homeostasis is the balance between the level of cell death and the level of proliferation. Two important hallmarks of tumorigenesis can cause disturbance of this balance; deregulation of the proliferative signaling pathway and deregulation of the apoptotic pathway [88, 89]. Michael-Robinson *et al.* previously reported on a cohort of 100 colorectal cancer patients, in which they determined an ‘AI:PI ratio' [90]. This Apoptotic Index:Proliferation Index was based on M30 IHC for the apoptosis level and Ki67 IHC for the proliferation index. They were able to relate their AI:PI index significantly to patient outcome. Preliminary data from our center has also shown a better prognosis for patients with high levels of proliferation and apoptosis, especially in right-sided tumors (Reimers MS, Zeestraten ECM *et al.,* in progress). Therefore, further studies also need to be performed, which will focus on apoptosis as well as proliferation.

### Ki67

Proliferation is one of the most important hallmarks tumor cells must acquire for tumorigenesis [78]. The proliferation activity of a tumor can be estimated by determining the expression levels of specific cell cycle-related antigens by using IHC. A widely used marker is the ki67 antigen, which is expressed in the nuclei during all cell cycle phases, except during the G_0_ phase [91]. High expression levels of ki67 have been shown to correlate with patient outcome in many types of cancers, such as breast cancer, malignant lymphomas and astrocytomas [92, 93]. However, in colorectal cancer, there are discrepancies in the association between ki67 and prognosis and survival [94–96]. Most studies in CRC have reported an inverse relationship between ki67 expression and patient outcome; thus patients with high expression of ki67 in their tumor sections showed a better chance of survival [76, 90, 94, 96]. Discrepancies still exist and the reasons for these remain unclear [94, 96, 97].

If we consider the balance between the level of cell death and the level of proliferation again, as previously mentioned, contradicting results between the different studies could be the result of differences in apoptosis in the tumor tissues, which were not evaluated in these ki67 studies simultaneously. High proliferation might be associated with survival advantages because these cells also undergo apoptosis resulting in tissue homeostasis. Michael-Robinson *et al.* showed that there was a significant correlation between the apoptotic index and proliferation index, indicating some degree of co-ordinated regulation [90]. However, a high ki-67 index was associated with improved survival in MSI tumors only and therefore microsatellite status might influence ki67 expression as well. Since most MSI tumors are found on the right side of the tumor, location of the tumor might also influence ki67 expression [98]. Other studies on ki67 did not stratify for microsatellite status or location of the tumor [76, 94, 96]. In conclusion, contradictory results exist regarding ki67 expression. Further research should focus on combined analysis of proliferation and apoptosis, as a balance might exist between these two hallmarks of cancer. Furthermore, analyses should be stratified for microsatellite status and location of the tumor, in order to truly understand the prognostic value of ki67.

### Immune-related markers

Historically, the immune system has been attributed with an important role in controlling tumor growth and metastasis [99–101]. Evasion of immune surveillance and suppression of the immune system were therefore two important traits that cancer cells had to acquire during the process of tumorigenesis [102]. Research from the last century has indicated that the effects the immune system has on tumor cells, both in the tumor microenvironment as well as during the process of tumor metastasis, can also contribute to tumor progression [103].

The first marker of tumor-immunogenicity is the level of HLA class I expression on cancer cells. Tumor cells can escape cytotoxic T-cell (CTL) recognition through downregulation or complete loss of HLA class I, resulting in minimization of tumor-associated antigen (TAA) expression and subsequently less destruction of tumor cells by CTLs [100, 101]. HLA class I expression has been shown to be of prognostic value in several types of solid cancer [104, 105]. However, the results in CRC specifically have been contradictory [106–109]. Downregulation of HLA class I makes tumor cells more prone to natural killer (NK) cell destruction. Non-classical HLA-E and HLA-G also play an important role in immune surveillance by NK cells. Presence of HLA-E and HLA-G cause an inhibitory signal to NK cells, resulting in further immune escape [110–112]. Furthermore, immune reactivity can become suppressed by the attraction of immunosuppressive regulatory T cells (Tregs) into the tumor microenvironment [113, 114]. The immunosuppressive effect of Tregs has been proven, with a high density of tumor-infiltrating Treg associated with an unfavorable prognosis in a wide range of human carcinomas, including breast and lung cancer [115, 116]. However, in colon cancer, different results were reported as well, with more Foxp3+ cells correlated with a better patient survival [117, 118].

Microsatellite instability has been shown to be characterized by a specific immune response [119]. Accumulation of frameshift-derived-peptides (FSP) may contribute to immune recognition and dense lymphocyte infiltration observed in MSI tumors [119]. However, these tumors grow out to large tumor masses as well, possibly due to loss or downregulation of HLA class I, also frequently observed in these tumors [120]. Furthermore, MSI tumors showed a high infiltration of Tregs [119]. T cell responses in patients with MSI CRCs are frequently directed against selected microsatellite instability-induced FSP, possibly creating more immune-mediated tumor rejection [119, 120]. Therefore, immune escape mechanisms may play a role in tumors characterized by microsatellite instability, and thus both features should be considered when analysing clinical prognosis in this tumor type.

Besides T cells, innate immune cells also orchestrate an inflammatory environment that might inhibit or promote CRC development and progression, such as macrophages. Macrophages are a primary source of secreted pro-inflammatory cytokines, which are able to influence and stimulate growth and migration of tumor cells. For example, IL-6 released by macrophages directly promotes CRC cell progression. Furthermore, the interaction between IL-6 and IL-10 also influences CRC progression and prognosis by manipulating their microenvironment for tumor growth facilitation [121].

The interaction between tumor cells and immune cells is complex and multifaceted. As shown by our previous studies in breast cancer, immune markers are related to each other [104, 122]. In our opinion, studies based solely on one immune marker are not sufficient. Therefore, more studies need to be performed that focus on combining immune markers. Also, contradictory results from previous studies need to be studied further, also taking into consideration microsatellite instability.

### Genomic signatures

The recognition that molecular features of cancer—including gene expression profiles—are connected to clinical outcome has led to the development of molecular tests that provide important prognostic and predictive information to aid clinical decision making. Genomic Health Inc. (Redwood City, CA, USA) has developed four studies in stage II and stage III colon cancer, involving more than 1800 patients in total, where genomic profiling has identified genes that are predictive of recurrence in resected colon cancer patients who were treated with surgery alone or surgery + 5-FU/LV chemotherapy [123]. The results from these studies enabled the design of the 12-gene colon cancer Recurrence Score, which was then validated in a large, independent, prospectively designed study in stage II colon cancer patients from the QUASAR clinical trial. In the QUASAR validation study, the Oncotype DX® Colon Cancer Assay (the colon cancer Recurrence Score) was validated as a predictor of risk of recurrence in stage II colon cancer patients following surgery [124]. The Recurrence Score predicted recurrence risk independently of pathologic T stage, tumor grade, number of nodes examined, lymphovascular invasion and microsatellite status, providing information not captured by the existing markers used in clinical practice. The Recurrence Score thus addressed individualized recurrence risk information needed for optimal treatment planning in stage II colon cancer. Since January 2010, the Oncotype DX® Colon Cancer Assay has been offered by Genomic Health's clinical laboratory under Clinical Laboratory Improvement Amendments (CLIA) standards for clinical use and is now available to support treatment planning for stage II and stage III colon cancer patients [125].

Furthermore, ColoPrint® (Agendia, Amsterdam, The Netherlands) also showed promising results [126]. In this study a prognostic 18-gene signature was identified on the basis of unbiased gene selection, searching the whole genome for genes that had the highest correlation to a tumor relapse event. The signature was validated in an independent set of 206 patients with UICC stage I–III colon cancer from Barcelona, Spain and in 135 clinical samples of patients with stage II colon cancer from Munich, Germany, using a diagnostic microarray platform.

Previous attempts have also been made to correlate gene expression profiles with recurrence in stage II and -III colorectal cancer [127–130]. However, these studies have generally used fresh frozen tissues, which are less applicable in clinical practice and have addressed small patient cohorts and therefore lacked statistical power for convincing evidence.

Genomic signatures potentially have a high prognostic value and some are already in use in clinical practice, like Oncotype DX®. Other genomic signatures need to be validated before introducing them in clinical practice, preferably using tissues from randomized clinical trials.

### PIK3CA

Activation of the phosphatidylinositol 3-kinase (PI3K)/AKT pathway has been associated with the development of a number of human cancers, including CRC [131]. The PIK3CA gene encodes the p110 alpha catalytic subunit of PI3K [132]. Mutations in this gene have been identified in CRC, with most mutations localized in exon 9 and 20 [133]. Mutations have been shown to activate the AKT-pathway, driving cell proliferation, and are present in 10–30% of all CRCs [134]. PIK3CA mutations were related to a worse chance of survival in CRC patients [135, 136]. However, only a mutation in exon 20 might be responsible for this worse chance of survival [137]. Also, when stratified by KRAS status, a worse colon cancer-specific mortality associated with a PIK3CA mutation was only found in KRAS wild-type tumors [136].

### 18q Loss of heterozygosity

Allelic loss of 18q has been thought to occur late in the process of carcinogenesis and occurred in approximately 70% of CRCs. ‘Deleted in colon cancer' (DCC), SMAD4 and many other important candidate genes have been identified on 18q [134]. Patients who harbored an 18q loss of heterozygosity (LOH) showed a worse OS [138, 139], but other studies showed contradictory results [140, 141]. Jen *et al.* showed that stage II and -III patients with an intact 18q had a significantly better 5-year OS, compared with patients with allelic loss of 18q, suggesting a prognostic role of 18q LOH [138]. A meta-analysis also showed that patients with 18q allelic imbalance and DCC loss of expression were associated with a worse survival, compared with patients with an intact 18q and expression of DCC [134].

Unfortunately, some studies did not account for MSI status, which seemed to influence the association of 18q LOH with survival [47, 142]. The prognostic effect of 18q LOH was lost in multivariate analysis in these studies when accounted for MSI status. Therefore, the prognostic value of 18q LOH remains unclear. Validation is warranted to draw further conclusions.

### CIMP

In the last few years, the existence of a new pathway for CRC pathogenesis has gained attention, which involves the transcriptional silencing of tumor suppressor genes by hypermethylation of CpG islands of the promoter region of various genes [143]. These tumors are classified as having the CpG island methylator phenotype (CIMP) [144]. One-third to one-half of all CRCs may evolve through this pathway [145]. CIMP tumors with methylation-induced silencing of MLH1 constitute the majority of sporadic MSI CRCs [146].

However, most CIMP-positive tumors are associated with microsatellite stability (MSS). These CIMP MSS tumors are comparable with MSI CRC on certain clinical and pathological features, including a predilection for females, advanced age of disease onset, predilection for proximal colon, poor differentiation and mucinous histology [147]. Jover *et al.* showed that CIMP did not influence disease-free survival (DFS) and that patients with CIMP-positive tumors did not benefit from 5-FU-based adjuvant chemotherapy [147]. On the contrary, CIMP positive CRCs showed a worse overall survival after surgery alone. However, the same study reported that CIMP-positive CRCs showed a better response to the combination of surgery and 5-FU treatment, which could be caused by aberrations in folate- or methyl group metabolisms in CIMP positive tumors [148]. Taken together, these studies might support that CIMP could be used as a prognostic marker, but further research is necessary to confirm and validate these data.

### Chromosomal instability

In addition to microsatellite instability and CIMP, the chromosomal instability (CIN) pathway is also involved in colorectal cancer pathogenesis. Most CRCs arise through this pathway, which is characterized by widespread imbalances in chromosome number (aneuploidy) and loss of heterozygosity [149]. CIN is observed in 65–70% of sporadic colorectal cancers. Defects in chromosomal segregation, telomere stability and the DNA damage response have been described, but the complete mechanism of CIN remains unclear [149].

The CIN phenotype was associated with a less favorable outcome for patients, compared with tumors with MSI. Patients with CIN tumors showed a decreased overall and progression-free survival, compared with patients with MSI tumors, irrespective of ethnic background, anatomical locations and adjuvant treatment with 5-FU [150]. In large meta-analyses the prognostic value of CIN has been established with a HR of 1.45, compared with CIN-negative tumors [151].

In the future, the mechanisms that initiate CIN and the relationship between CIN and tumor progression need to be better defined in order to implement CIN as a biomarker in clinical practice.

## PREDICTIVE BIOMARKERS IN CRC

The predictive markers in this review are divided in therapy-related predictive markers; chemotherapy- and aspirin-related; and predictive markers for treatment toxicities in CRC patients.

### Therapy-related predictive biomarkers

#### Microsatellite instability

In addition to the positive prognostic influence of MSI in CRC, a predictive role for microsatellite status has been demonstrated by using data from randomized clinical trials of 5-FU-based therapy versus surgery-only control [152, 153]. In these trials, treatment differed by MSI status and patients with MSI-high tumors who were treated with 5-FU-based therapy had a trend towards a worse outcome compared with surgery-alone controls. In contrast, other studies reported similar outcomes for MSI-high patients with chemotherapy [154] or even showed a greater benefit from 5-FU-treatment [36, 155, 156]. These contradictory results could be explained by the differences in study design, as these latest studies included patients who were not randomly assigned to 5-FU therapy versus control, thus allowing selection bias or other limitations inherent to non-randomized studies. Also, Sinicrope *et al.* reported a positive reduction in disease progression rate in MSI CRC patients treated with 5-FU, but this was only due to the HNPCC cases [36]. Therefore, these cases need to be separated from the sporadic MSI cases in further studies.

Establishing microsatellite status could be of particular interest for stage II patients, where the modest therapeutic effect of 5-FU-based therapy (2–4% in 5-year DFS) emphasized the need for prognostic and predictive markers to risk-stratify these patients [157, 158]. The favorable prognosis of MSI CRC patients and the lack of benefit from 5-FU based therapy in patients with MSI tumors support a non-adjuvant treatment approach. Therefore, if we could establish the predictive value of MSI in these patients, a lot of patients could be spared from over-treatment, expenses, treatment-related toxicities and reduced quality of life during 5-FU-treatment.

Unfortunately, patients included in the above-mentioned studies were treated 20–30 years ago in multiple countries. The current standard for adjuvant therapy in CRC has changed over time. The current standard for stage III CRC nowadays is infusional fluorouracil, leucovorin and oxaliplatin. Preliminary data suggest that adding either oxaliplatin or irinotecan to 5-FU/leucovorin may overcome possible MSI resistance to 5-FU treatment and thus even alter the predictive value of MSI [159, 160]. However, these recent data need further investigation and the data so far available do not justify excluding patients with stage III disease and MSI tumors from treatment according to current regiments.

#### KRAS

A randomized clinical trial conducted by the National Cancer Institute of Canada Clinical Trials Group (NCIG CTG) in collaboration with the Australasian Gastro-Intestinal Trials Group (AGITG) showed that, among CRC patients who did not respond to advanced chemotherapy, monotherapy with cetuximab—a monoclonal antibody directed against the epidermal growth factor receptor (EGFR)—improved their overall survival and progression-free survival and preserved their quality of life in comparison to best supportive care alone [161]. Cetuximab and panitumumab are registered for CRC patients whose tumors express EGFR protein, as determined by immunohistochemistry. However, it has clearly been demonstrated that this method has no predictive value in terms of cetuximab activity in colorectal cancer, since there was no tendency towards a higher response rate with higher EGFR expression [162, 163]. Furthermore, resistance to this treatment is common and might be explained by KRAS*.* KRAS can acquire activating mutations in exon 20 resulting in isolation of this pathway from the EGFR effect and thus rendering EGFR inhibitors, like cetuximab, ineffective [164–168]. Indeed, previous studies showed the ineffectiveness of cetuximab or other EGFR inhibitors for CRC patients bearing mutated KRAS [164–167]. Therefore, treatment of CRC patients with cetuximab, with all its costs and toxicities, would be most appropriate for CRC patients bearing wild-type KRAS only. Furthermore, the addition of EGFR-antibodies to chemotherapy for patients with KRAS mutations appeared to be detrimental [169]. KRAS mutation has thus emerged as the major negative predictor for EGFR therapy efficacy, resulting in clinical recommendation for use by patients with wild-type KRAS tumors only [170]. KRAS mutational testing of metastatic CRC has become routine and is nowadays incorporated in many centers. However, not all patients with KRAS wild-type tumors benefit from cetuximab and panitumumab and the positive predictive value is low, with a sensitivity of 47%. Additional markers are necessary to better identify which patients will benefit from EGFR therapy. Less-frequently observed KRAS mutations beyond the well-studied codons 12 and 13, mutations in NRAS, BRAF and PIK3CA also showed that they are associated with resistance to anti-EGFR therapy [170].

#### BRAF

As described above, treatment decisions on cetuximab solely based on KRAS, with an occurrence of only 30–40% in non-responsive patients [166, 167, 171, 172], might not be adequate. Therefore, the identification of additional markers of EGFR-targeted therapies in CRC is greatly needed. Since EGFR triggers two main signaling pathways—the RAS-RAF-MAPK axis and the PI3K-PTEN-AKT pathway—resistance to anti-EGFR therapy could also be caused by other members of these pathways, like BRAF as part of the RAS-RAF-MAPK pathway [165]. BRAF is the principal downstream effector of KRAS [173, 174]. Only a few studies have been performed on the relationship between BRAF and the effect of cetuximab, both showing that BRAF mutations were related to resistance for EGFR-targeted therapies [62, 63]. Although evidence is still insufficient to demonstrate a real association of BRAF mutations with non-responsiveness to anti-EGFR therapy, it has been recommended by the National Comprehensive Cancer Network (NCCN) for this purpose. Combined analysis of both KRAS and BRAF could be used to select patients eligible for EFGR-targeted treatment, with evident medical and economic implications. Further molecular markers are needed and more studies, especially a randomized controlled trial, need to be performed in order to confirm these results. Preliminary data suggest that the ineffectiveness of EGFR-targeted therapies could be restored by adding a BRAF inhibitor, sorafenib, concomitantly with cetuximab or panitumumab [63]. This treatment combination is currently undergoing clinical assessment in CRC in a trial sponsored by the National Cancer Institute (NCT00326495) and might be a promising discovery, but also requires further investigation. In addition to sorafenib, other compounds targeting either BRAF (PLX4032 and PLX4720) or its downstream effectors (ARRY-162, AZD6244 and PD0325901) are in clinical development and could be exploited in combination with EGFR-targeted therapy [175]. PLX4032 is a V600 BRAF inhibitor which has shown promising results in melanoma. However, in CRC the clinical activity was modest, with only a 5% response rate. On the other hand, PLX4720 caused substantial delays in tumor growth, including tumor regression, without toxicities [176].

### COX-2

Currently, the use of aspirin is gaining interest in CRC treatment. Aspirin and other non-steroidal anti-inflammatory drugs (NSAIDs) have been shown to be effective in preventing colorectal cancer [177–179]. Aspirin inhibits cyclooxygenase-2 (COX-2), which is expressed in 70% of colorectal tumors and increases with a more advanced stage of the disease [180, 181]. COX-2 plays an important role in colorectal carcinogenesis, invasion, angiogenesis and metastasis. Several studies have shown that selective COX-2 inhibitors are able to reverse this COX-2 effect [182]. Recent studies showed that aspirin might also play a role as adjuvant treatment in CRC [183, 184]. Chan *et al.* showed that regular use of aspirin after a CRC diagnosis is associated with a lower risk of colorectal cancer-specific and overall mortality, especially for individuals with tumors that overexpress COX-2 [180]. Also, the same group reported that aspirin reduced the risk of CRC exclusively for individuals with elevated COX-2 expression [185]. Though these findings were from observational studies, they confirmed experimental data that prostaglandins and non-prostaglandin COX-2 products are central to the pathogenesis of CRC. They are also in accordance with animal studies, in which genetically modified mice had defective APC-genes and in which rats had CRC after administration of exogenous carcinogens [186]. Elevated COX-2 expression in genetic APC deficiency was related to enhanced tumorigenesis, while deletion of the COX-2 gene had the opposite effect [187, 188]. These data strongly suggest a central role of COX-2 in CRC and their inhibition as an effective chemopreventive measure. Unfortunately, studies investigating COX-2 expression for patients treated with aspirin are scarce, prompting the need for further validation of this possible biomarker.

Recent studies showed that aspirin not only influences COX-2 expression, but COX-1 inhibition might contribute to the anti-tumor effects of aspirin as well, for example at low-dose aspirin [189]. Experimental evidence also suggests additional COX-independent actions of aspirin and other NSAIDs, such as modifications of transcription factors (NFκB), induction of apoptosis and DNA stabilization [189].

Furthermore aspirin use, even at low doses appropriate for cardiovascular risk management, is not without risks and roughly doubles the incidence of gastric bleeding [190]. These drugs have been shown to enhance cardiovascular risks as well [191]. Appropriate biomarkers are therefore needed to improve risk–benefit ratio. Since the exact mechanism of aspirin is not yet known, COX-2 tumor expression is not ready to be used as a biomarker to select CRC patients for aspirin treatment.

### PIK3CA

In addition to the influence of tumor COX-2 expression on aspirin treatment benefit in CRC as detailed above, a recent study showed that only CRC patients bearing a mutation in PIK3CA (exon 9 or exon 20) benefitted from aspirin treatment, and not patients with wild-type PIK3CA tumors [192].

The phosphatidylinositol 3-kinase (PI3K) signaling pathway plays an important role in carcinogenesis [133]. Mutations in PIK3CA are present in 15–20% of colorectal cancers [48, 193, 194]. Up-regulation of PI3K enhances COX-2 activity and prostaglandin E2 synthesis, resulting in inhibition of apoptosis in colon-cancer cells [195]. Aspirin may suppress cancer cell growth and induce apoptosis by blocking the PI3K pathway [196].

Unfortunately, only one study on the role of PIK3CA mutations with aspirin treatment in CRC has so far been performed, which also had limited statistical power [192]. More studies are needed to validate these results and to unravel the therapeutic effect of aspirin in CRC.

### Toxicity-related predictive biomarkers

#### DPD deficiency

Capecitabine, 5-fluorouracil (5-FU) and tegafur all belong to the fluoropyrimidines. Fluoropyrimidines are one of the most frequently used anti-cancer treatments in colorectal cancer, with a good tolerability for most patients. However, in approximately 5–10% of patients, severe toxicity arises after treatment has started, which can sometimes be life-threatening [197, 198]. The intolerance of fluoropyrimidines is often caused by dihydropyrimidine dehydrogenase (DPD) deficiency, which is present in approximately 4% of the western population [199]. In 80% of patients with DPD deficiency, the use of fluoropyrimidines in standard dose results in severe toxicity [200]. Screening for this intolerance could identify ‘at risk’ patients, resulting in fewer toxicity-related hospital admissions and lower medical costs. Also, treatment could be adjusted for these toxicities, with lower doses or dose titration according to arising toxicities. Titration of the dose in DPD-deficient patients could significantly reduce the frequency of severe, potentially deadly toxicity caused by fluoropyrimidines [201].

DPD deficiency can be determined by ‘real-time’ PCR, which is a simple technique and only requires 1 ml of blood with a sensitivity and specificity of 100% [202]. Unfortunately, current genotyping of DPD deficiency only detects 25–50% of all DPD-deficient patients, as only DPYD*2A can so far be detected, which has a frequency of 1–2% in the total population [203]. New mutations have been identified, which are related to fluoropyrimidine toxicity, such as DPYD 2846A > T and 1236G > A, and could be implemented in genotyping DPD deficiency [203, 204]. In clinical practice, the lower toxicity associated with modern infusional or oral 5-FU-based regimens make it impossible to screen the entire population for 30 polymorphisms associated with DPD deficiency. Despite the clear effect on toxicity, the prognostic and predictive value remains unclear, with studies reporting contradictory results. Possibly clinicians responded differently on the toxicities encountered. Despite well-investigated evidence, the pharmocogenetic basis of varied DPD activity needs further investigation [151].

#### UGT1A1 polymorphism

Irinotecan is a topoisomerase I inhibitor that interrupts DNA replication in cancer cells, resulting in cell death [205, 206]. The prodrug irinotecan is activated by carboxylesterase to the active metabolite SN-38, which is 100–1000 times more cytotoxic than the parent drug [205]. SN-38 is further catalyzed into the inactive glucuronide derivative SN-38G by several hepatic and extrahepatic UGT enzymes. One of the major isozymes involved in this catalyzation is UGT1A1 [207]. A decrease in the level of functional UGT1A1 enzyme reduces a person’s ability to metabolize SN-38 to an inactive form, and is also associated with a higher risk of adverse side effects, like neutropenia and diarrhea caused by high levels or prolonged exposure to the active form [208, 209]. At least 63 UGT1A1 variants have been described, including single base pair changes, frame shift mutations, insertions and deletions in the promoter region, five exons and two introns of the gene. Most variants are associated with an absent, reduced or inactive enzyme; one is associated with an increased enzyme level and the effects of some variants are unknown [210]. Although several clinical trials have confirmed that patients carrying different genotypes of UGT1A1 had varied degrees of tolerance to irinotecan, it is still unclear whether UGT1A1 has any influence on treatment efficacy. Three studies examined the impact of UGT1A1 isoforms on treatment outcome; however, their conclusions were inconsistent [211–213]. Many western studies have suggested that UGT1A1*28 is significantly associated with irinotecan-induced toxicity [214–216]. In particular, patients bearing UGT1A1*28 (TA7/7) had a high possibility of developing severe neutropenia and diarrhea. Based on this, doctors are warned that patients with UGT1A1*28 (TA7/7) should start with a reduced dose of irinotecan, although the details on how to adjust the dose have not been specified [210]. On the other hand, research in Asian countries has shown a lower incidence of UGT1A1*28 (TA7/7), while UGT1A1*6 (A/A) is more often found and may replace UGT1A1*28 as a key regulator in UGT1A1 expression [217, 218].

Palomaki *et al.* stated a few problems regarding the use of UGT1A1 in clinical practice; there seems to be a clear relationship between UGT1A1 genotype and severe neutropenia, but there is no direct or indirect evidence to support the clinical utility of modifying an initial and/or subsequent dose of irinotecan for patients with metastatic CRC as a way to change the rate of adverse drug events. Also, the data on the clinical validity of tests for UGT1A1 variants other than *28 are limited and the analytic validity of UGT1A1 testing in clinical practice is unknown. Laboratories offering such testing may include variants in addition to *28, for which little evidence is available. Furthermore, there are limited data on UGT1A1 variants in Hispanic and African-American populations. In order to recommend UGT1A1 testing in clinical practice, additional studies are needed, in order to understand the potential effects of alleles that are rare for Caucasians but more common for other racial/ethnic groups and studies should focus on all variants of clinical significance in the population [210].

## DISCUSSION

Our knowledge of the process of tumorigenesis has been increasing dramatically in the past decades. As postulated by Hanahan *et al.*, cancer cells must acquire biological capabilities during the multi-step development of human tumors. Sustaining proliferative signaling, evading growth suppressors, resisting cell death, enabling replicative immortality, inducing angiogenesis, activating invasion and metastasis, reprogramming of energy metabolism and evading immune destruction are all hallmarks of tumorigenesis [78]. Recognition of these concepts will increasingly affect the development of new treatment modalities in human cancer. Currently, recognition of these concepts has led to the identification of a lot of biomarkers, which might be of prognostic or predictive value in CRC.

Identifying and understanding molecular markers can improve the effectiveness of treatment in several ways; it may lead to the development of marker-specific therapies and it may also improve the selection of adjuvant therapies by identifying those patients who will benefit most and therefore avoid toxic side effects with the least risk of recurrence. The use of biomarkers might also have influence on socio-economic questions, decreasing the economic burden.

In this review we demonstrated the high potential of well-studied prognostic and predictive biomarkers in CRC. Only mutant KRAS, mutant BRAF, MSI and the Oncotype DX® Colon Cancer Assay are currently used in clinical practice for determining whether to treat metastatic CRC patients with cetuximab or panitumumab, for the evaluation of Lynch syndrome and to inform treatment planning in stage II and -III colon cancer patients. Implementation of these biomarkers, however, has been beneficial: for example, screening for MSI has resulted in increased identification of patients with Lynch Syndrome [219].

Unfortunately, other biomarkers are not ready to be introduced into clinical practice, which can be explained by several factors. Firstly, study characteristics of the individual investigations on biomarkers varied widely. Sometimes a marker with prognostic significance was demonstrated, but only in a highly selected group of patients. Secondly, well-standardized protocols to detect the biomarker were not applied for any of the markers, particularly IHC. Also, there seems to be no standardized method for quantification of the expression level of a particular biomarker. Lack of consensus in performing studies may greatly influence the interpretation of the results of these studies. If studies are not performed according to standardized protocols, it is extremely difficult to compare the results of the individual studies. The handling of tissues has been well recognized as contributing to assay variability as well as issues in assay validation [24]. Some tissues are amenable to repeated sampling, without concern over substantial tissue heterogeneity or sampling issues, but often tissue-preserving methods cause damage or even destruction of tissues. New assays make great demands on the tissues but it is impractical to replace the current tissue-handling methods entirely. An integrated approach to the development and validation of integral biomarker assays might solve this problem. The difference between how a biospecimen is handled in a clinical setting and in a research setting must be reduced [24]. Thirdly, none of these biomarkers are validated in larger cohorts or even in prospective trials. Previously, a five-step program for the introduction of biomarkers in clinical practice was developed, with the first step being biomarker development in a pre-clinical, exploratory setting, followed by verification of this biomarker in a large retrospective study, validation and finally confirmation in a prospective, randomized, controlled trial [25]. Unfortunately, this program has not so far been implemented, which might explain why biomarkers are used so rarely in daily practice. Furthermore, most studies did not consider tumor heterogeneity, the influence of tumor–stromal interaction and the percentage of tumor in a sample, which also might influence results gained from molecular or immunohistochemical analyses. Finally, using only one marker to predict the outcome of patients seems inappropriate as, according to Hanahan *et al.*, tumor cells acquire multiple capabilities for tumorigenesis [78]. Our group has recently demonstrated that patients with both presence of HLA class I expression and Treg tumor infiltration had fewer relapses when treated with chemotherapy [220]. Combining markers might add more clinical value and gain more information about tumor aggressiveness.

In conclusion, the use of molecular markers and other biomarkers in CRC allows the identification of genes and biomarkers, which might predict individual prognosis and recurrence rate. Also, it might optimize treatment results and minimize treatment toxicities, resulting in a decrease of economic burden and eventually the use of precision medicine in treating CRC patients. Only a few biomarkers are currently used clinically. However, in order to introduce more biomarkers in clinical practice, future studies need to consider the combination of markers, standardizing protocols and avoiding selection bias. Furthermore, simple, cheap, automated and standardized assays for the detection of molecular markers are necessary. Most importantly, results need to be validated in larger studies, followed by prospective trials.

**Conflict of interest:** none declared.
